# Permutation Entropy as a Measure of Information Gain/Loss in the Different Symbolic Descriptions of Financial Data

**DOI:** 10.3390/e22030330

**Published:** 2020-03-13

**Authors:** Jan Kozak, Krzysztof Kania, Przemysław Juszczuk

**Affiliations:** Faculty of Informatics and Communication; Department of Knowledge Engineering, University of Economics, 1 Maja 50, 40-287 Katowice, Poland; kkania@ue.katowice.pl (K.K.); przemyslaw.juszczuk@ue.katowice.pl (P.J.)

**Keywords:** forex market, permutation entropy, symbolic analysis, symbolic data

## Abstract

Financial markets give a large number of trading opportunities. However, over-complicated systems make it very difficult to be effectively used by decision-makers. Volatility and noise present in the markets evoke a need to simplify the market picture derived for the decision-makers. Symbolic representation fits in this concept and greatly reduces data complexity. However, at the same time, some information from the market is lost. Our motivation is to answer the question: What is the impact of introducing different data representation on the overall amount of information derived for the decision-maker? We concentrate on the possibility of using entropy as a measure of the information gain/loss for the financial data, and as a basic form, we assume permutation entropy with later modifications. We investigate different symbolic representations and compare them with classical data representation in terms of entropy. The real-world data covering the time span of 10 years are used in the experiments. The results and the statistical verification show that extending the symbolic description of the time series does not affect the permutation entropy values.

## 1. Introduction

In the age of information, the main difficulty is not to obtain data, but rather to extract the most important and, at the same time, the non-redundant information. In the field of finance, where the high-frequency trading systems are currently responsible for most of the transactions, it is crucial to obtain the information allowing achieving the best results in a very short time [1,2].Decision-makers never before had so many different investment opportunities; yet, the decision process was never before so complicated and risky. While the time needed to make a decision is shortened, more noise is observed. This is especially visible in the case of very volatile instruments like cryptocurrencies or the foreign exchange market [3]. Especially in the second case, where concepts like leverage are commonly used, it is very easy to lose a large amount of money in a very narrow time window.

Developing new techniques and measures resembles the trial and error method. Every new proposition is tested on historical data with the assumption that the same modus operandi will work for the future. In opposition to this approach, our proposition is to measure the method with entropy to answer what is the gain/loss of information question compared to another one. By introducing measures and indicators, we would like to see and understand this system. As for every measure or indicator, we can calculate its own entropy; we can further compare different methods and indicators using its entropy to answer the question of which of them carries more or less amount of information about the primary system (an instrument on the market).

The set of tools available to the decision-makers is growing constantly almost every single month. Concepts related to technical analysis [4], fundamental analysis [5], social trading [6], and different data presentations are extended by new propositions. Despite the undeniable simplicity of the technical analysis and other simplifications of the financial data representations, decision-makers still struggle with the difficulty of the analysis for the single financial instruments. Many non-linear dependencies, random noise, and hidden variables make it difficult to efficiently analyze the market situation. The problem is exacerbated when a decision needs to be made in seconds. It should be noticed that the smaller the time window, the higher the share of noise in the total information observed.

Among the methods dealing with the noise reduction in financial data are various technical indicators, combinations of market indicators, and symbolic representation of the data. The last approach is especially useful in the case of decision support systems used to aid the traders in the decision process. However, one of the main drawbacks of such an approach is a visible limitation of the information delivered to the decision-maker. For instance, Japanese candlesticks visualizing high-low-close-open prices are one of the most popular charts used in the technical analysis. In such a context, the most basic linear chart including only the “close” price of the instrument can be understood as a more simplified symbolic representation of the market than Japanese candlesticks. Renko or Heiken Ashi charts are also in the category of the simplified price representation. The opposite side includes concepts like Ichimoku charts [7], which extend the information derived for the decision-maker and can be used as a confirmation signal. Other technical indicators fall in the same category, where some additional information is added for the decision-maker, while the simplification of the initial price chart is rarely seen.

Each indicator has a different informative value for the investor, and their selection is more a matter of individual preferences than an objective assessment of effectiveness. In the article, we suggest using entropy as an objective measure of the amount of information that various indicators and different ways of describing the time series carry. Because there are dozens of different ways to describe time series and dozens of different indicators, in this article, we discuss the narrower problem of symbolic representation of the data based on the relative and discretized price values. It is commonly assumed that the symbolic representation derived for the decision-maker in such a way leads to the limitation of the observed information. Intuitively, extending symbolic representation and adding new information to a previously generated symbolic chart should increase the amount of information obtained. We use the entropy concept to verify experimentally if the above statement is true. The novelty of the approach consists of treating entropy directly as a measure of the amount of information provided to the recipient (in our case, on the example of an investor in the forex market).

In general, we focus on using the entropy-based approach to investigate, what is the information loss/gain between the symbolic financial data representation and the original financial data on the forex example. We discuss the concept of the symbolic data and briefly recall our proposed representation. Further, we examine whether the size and construction of symbolic data used to describe the market situation affect entropy values and thus indicate a different amount of information obtained for investors.

All the above goals are verified within the numerical experiments section including the statistical tests. These experiments are preceded by the methodological description including the data transformation, as well as the entropy concept details. To the best of the authors’ knowledge, the permutation entropy was not used as a tool to measure the information gain from the symbolic data on the market. At the same time, it is worth noting that the numerical experiments, as well as the statistical verification show that the extension of the symbolic representation does not visibly affect the entropy values.

The article is organized as follows: In the next section, we present the theoretical background of the research. The literature review is discussed as well. The third section is focused on the various symbolic data representations. The fourth section includes the numerical experiments and the statistical verification of the generated results. The discussion is included after the numerical experiments. Two last sections include the short summary and details of future research.

## 2. Theoretical Background

Entropy can be treated as a measure of the complexity of time series in a variety of fields. Today, we observe growing interest in using entropy in various areas and a growing number of publications related to measures of complexity and entropy in particular. Among many propositions, permutation entropy (PE) introduced by Bandt and Pompe [8] was designated to investigate time series. The advantages of PE have made it widely used, and the modifications proposed in the literature increased its usefulness in new areas of research.

In our research, we adopt an enhanced time-dependent pattern entropy method introduced in [9] (see also [10,11]) that reduces variations to binary symbolic dynamics and considers the pattern of symbols in a sliding temporal window.

Permutation entropy as a time series complexity measure belongs to the wider family of ordinal and symbolic methods. The main feature of ordinal methods is that they do not use the actual values of time series $\left(x_{1},x_{2},\ldots ,x_{N}\right)$, but the information about relations $x_{i}<x_{j}$ or $x_{i}>x_{j}$ with nearest or distant neighbors. Because permutation patterns can have different lengths, the parameter *n* is set. Next, a set of N−n+1 overlapping vectors is built $\delta X\left(i\right)=\left[x_{i},x_{i+1},\ldots ,x_{i+n-1},i=1,\ldots ,N-n+1\right]$. Next, values in each vector are permuted in increasing order:

(1) $$\{x_{i+k_{1}-1}\leq x_{i+k_{2}-1}\leq \ldots \leq x_{i+k_{n}-1}\leq k_{1},k_{2},\ldots ,k_{n}\leq n\}.$$

Finally, each vector is described by the permutation pattern $\left[k_{1},k_{2},\ldots ,k_{n}\right]$ of *n* symbols {1,…,n}. The relative frequency of each permutation pattern is calculated as follows:

(2) $$p\left(\pi \right)=\frac{Q(\pi )}{N-n+1},$$

where Q(π) is the number of occurrences of the pattern π. Permutation entropy of order n≥2 is defined as:

(3) $$H\left(n\right)=-\sum_{i=1}^{n!}p\left(\pi_{i}\right)\cdot log\left(\pi_{i}\right),$$

where for information entropy, a base = 2 for log is assumed.

For example, for a given time series {6,8,5,7,4} where N=5 and n=3, we have 5−3+1=3 permutation patterns: {312} as $x_{3}=5<x_{1}=6<x_{2}=8$, {231} as $x_{2}=5<x_{3}=7<x_{1}=8$, and again, {312} as $x_{3}=4<x_{1}=5<x_{2}=7$. Hence, $Q(\pi_{1})=1$, $Q(\pi_{2})=2$, and $p\left(\pi_{1}\right)=\frac{1}{3}$, $p\left(\pi_{2}\right)=\frac{2}{3}$, which is:

(4) $$H\left(n\right)=-\frac{1}{3}\cdot log\left(\frac{1}{3}\right)+\left(-\frac{2}{3}\cdot log\left(\frac{2}{3}\right)\right)\approx 0.528321+0.389975\approx 0.918296.$$

H(n) is bounded in [0,log(n!)]. Thus, H(n)=0 when the series is strictly monotonic, and the upper bound indicates completely random series. In general, higher PE indicates that the process described by the time series is more complex and unpredictable.

During subsequent research, additional parameters were added to the measure. The original assumption about comparing the nearest neighbors in permutation patterns was replaced with the second parameter: time delay [12,13,14] between neighboring equidistant time points that are to be compared.

Bandt and Pompe [8] recommended n=3,…,7. Later, in [12], C.Bandt claimed that for real-world time series, n>10 is not meaningful because of the fast growth of possible patterns. He recommended using *n* for which n! is smaller than the length of the series, even though the averaging effect of the entropy formula allows us to work with a larger *n*. Therefore, the value of *n* should be chosen so that N>>n! to allow every possible pattern of length *n* to appear in the series of length *N*.

PE has numerous advantages. PE is conceptually simple in the sense that it does not presuppose any model, and as a consequence, it has a minimal set of parameters. PE is invariant to nonlinear monotonous transformations, and in comparison to other measures, permutation entropy does not require a time series with a large number of elements [11,12]. From a technical point of view, PE is very easy to compute. In particular, it does not require any numerical optimization; it is computationally extremely fast and does not need preprocessing, which makes it suitable for big datasets [13]. Thanks to these advantages, PE has been applied in various domains. A comprehensive summary of permutation entropy itself and its applications can be found in recent surveys [15,16,17].

Permutation patterns offer also a new research tool, i.e., analysis of allowed and forbidden patterns. If the pattern does not appear in the time series, such a pattern is referred to as forbidden. The number of possible *n*-length patterns is known and is equal to n!. The number of forbidden patterns in relation to a total number of patterns $\frac{Fp}{n!}$ can be treated as a measure of system order because the lower the measure is, the more orderly and predictable the system is. The advantage of forbidden patterns analysis is that it can be used even for small datasets as if particular patterns appear frequently in the smaller dataset, the meaning of this fact arises. Due to this, it is used in financial time series analysis [18,19,20]. This dependency was also used in [21] for comparison of emerging and mature stock markets.

PE has also its limitations. PE takes into account only relations <, > and focuses only on the order of the elements in TS, and that makes PE “too rough” “too coarse-grained”) or too sensitive a measure for many applications. The values themselves are not taken into consideration, which suggests that PE is not focused on the degree to which the neighboring elements differ from each other.

**Figure 1:**
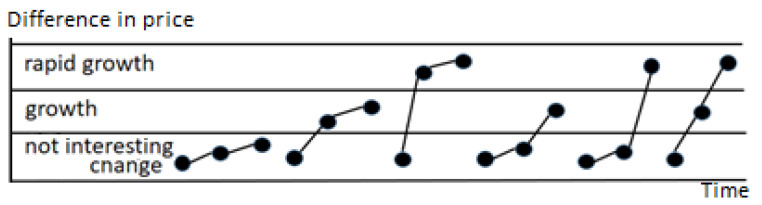
A few different patterns described by the same ordinal pattern {1,2,3}.

In Figure 1, a few patterns are presented. Although they represent different market situations and have different meanings for the user, they are all described by the same order pattern {1,2,3} and finally the same PE. Symbolic representation generated with the use of discretization (described in detail further in the next section) can be used to minimize the impact of the noise on the data. Thus, after initial preprocessing, the discretized values can be treated as the symbols in the time series.

To overcome this drawback, a few modifications have been proposed. Liu and Yue [22] proposed fine-grained permutation entropy (FGPE), which not only retains all the advantages and merits of PE, but also improves the performance for detecting the dynamical change of time series by introducing an additional factor to quantify the difference between the neighboring values. In such a case, the patterns identical from the point of view of PE could be further discriminated. They found that FGPE in opposition to PE is sensitive to peaks and sharp falls in time series. Other modifications of PE were proposed in [23,24].

Although PE is considered one of the best measures of the complexity of time series, it is worth mentioning that other types of entropy are also used. A comprehensive review of different entropy definitions and their application can be found in [25]. On the basis of PE, new measures are still invented like the “distance to white noise” proposed in [12].

Initially, entropy was used to study the dynamics of physical systems, but interest in using entropy in financial time series investigations grew particularly after 2008, when the financial crisis occurred, while the number of indicators did not signal any danger incoming. Since entropy is an indicator of complexity and unpredictability, in relation to financial variables, low entropy means it can be predicted, while high entropy indicates process randomness and high uncertainty. For this reason, financial time series are the subject of many entropy studies, and searching for financial risk indicators is still an urgent problem [26].

Many research works that used entropy for financial time series can be found ([27,28,29,30] for instance). Bentes and Menezes [31] used the concept of Tsallis entropy, which constitutes a possible generalization of the Boltzmann–Gibbs or Shannon entropy to investigate the volatility of seven indexes. It was also used in a comparative analysis of stock markets before the financial crisis in 1987 and 2008 [10]. A review of the application of entropy in finance can be found in [32,33,34].

Financial time series are characterized by small, but frequent and rapid changes of values that make them volatile, chaotic, multifractal, and temporally asymmetric [28]. However, these changes are small, and because of the leverage mechanism, commonly used on the financial market, they cause huge changes in investors’ portfolios. Values in these data are unbounded, and from a long-time perspective, they create trends and cycles [35]. Complexity, disorder, chaos, volatility, etc., of financial time series relate to uncertainty and risk. These are some of the most important factors influencing the behavior of investors on the market; hence the great interest in measures and methods that describe them.

## 3. Financial Data and Symbolic Representation

**Figure 2:**
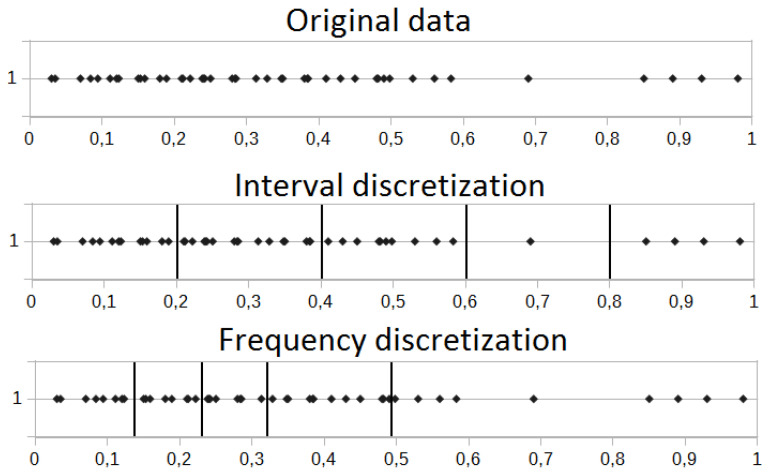
Comparison of the interval and frequency discretization process.

Original financial data are for most cases trend-based. Thus, the main problem is to estimate the potential price direction. This is true for the long-term investments; however, in the case of short-term, an approach excluding the noise present in the market and at the same time deriving the most important (and at the same time non-redundant) information is crucial. It is commonly known that the instrument price is not as important as relative differences between two neighboring values. Additional preprocessing transformation before moving into the symbolic representation is the discretization process. We took into account the frequency and interval discretizations. Both processes are introduced in Figure 2.

We investigated the relative changes of instruments; thus, the small changes near the zero value could be common in this case. This could lead to undesirable situations, where many small intervals for the frequency discretization near the zero value are generated. Moreover, the large price rise/drop is the most interesting case, which potentially leads to the signals on the markets. In that case, we are rather interested in equally-sized intervals derived by the interval discretization.

For most of the experiments, we used the relative data, which basically means that we used the information about the percentage price change between two successive readings. In such a case, most of the observed values were near the zero value (representing no change at all between two successive readings). Thus, the interval discretization dividing the analyzed interval was used. This allowed us to estimate the maximal and minimal relative price change and then divide this range into equal parts. The same situation could not be possible for the frequency discretization, where the vast majority of observations would be located near the zero value (small relative changes for the most cases). This could lead to the situation where the single discretization interval could be very small (for example between 0.1 and 0.15), while the other one could be too wide (like between 2.0 and 6.0). This situation is shown in Figure 2.

**Figure 3:**
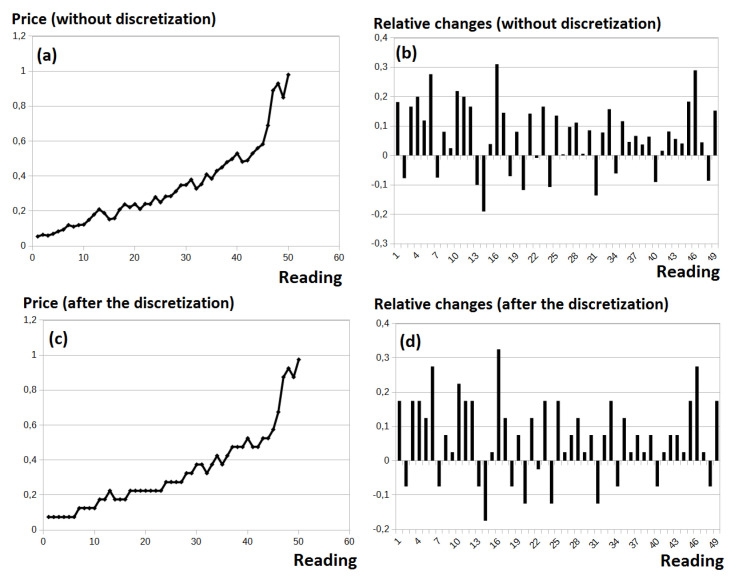
Different stages of the data preprocessing. (**a**) Original data; (**b**) discretization of the original data; (**c**) calculating the relative values; (**d**) discretization of the relative values.

Differences between the interval and frequency discretization are very small in the case of the original data, where the trend is included. This is due to the fact that the number of readings is rather uniformly distributed along the whole analyzed range (please see the Y axis in Figure 3a,c). To sum up, in this case, discretized data do not visibly differ greatly from the original data. However, in the case of the relative data, using the frequency discretization could lead to the situation where the data before the discretization and after the preprocessing are completely different. Our goal was to reduce the noise, where at the same time, the data representation was as close to the original data as possible.

As was emphasized, our biggest concern was the large noise observed on the market followed by the possibility of a trend occurring. This noise is observed in Figure 3a. Eventual discretization of the original data (presented in Figure 3b) does not solve the problem of excluding the trend. Thus, the discretization process presented in Figure 3d is followed by deriving the relative data from the original data (Figure 3c).

The relative data were achieved by the following formula:

(5) $$\Delta x_{i}=\frac{x_{i}}{x_{i-1}}-1,$$

where $x_{i}$ is the price value in reading *i*. There are several definitions of an asset return [36]. The asset return defined by (5) is called a simple return, while the continuously compounded return is defined as:

(6) $$r_{t}=ln\left(\frac{x_{i}}{x_{i-1}}\right)=ln\left(x_{i}\right)-ln\left(x_{i-1}\right).$$

In our approach, we used a simple return for the following reasons:

- a simple return is more frequently used by investors;
- according to Taylor’s formula $\frac{S(x+h)-S(x)}{S(x)}\approx log\left(\frac{S(x+h)}{S(x)}\right)$, the values calculated using both definitions are almost equal for small *h* and do not affect the final results;
- this definition is consistent with the definition of symbolic description used in further investigation.

Eventually, the symbolic time series *d* is built on the basis of past *k* readings:

(7) $$d=(\Delta \left(x_{i-k}\right),\Delta \left(x_{i-k+1}\right),\Delta \left(x_{i-k+2}\right),\Delta \left(x_{i}\right)).$$

Such a symbolic time series with the length of *k* is further examined on the basis of information derived for the decision-maker (this is obtained with the use of the permutation entropy).

In our approach, we also used the second concept of deriving relative changes related not to the difference between two neighboring price values, but rather between the first and $k^{\mathrm{th}}$ element in the time series. This leads to the following formula:

(8) $$d=\left(f\left(x_{i},x_{i-1}\right),f\left(x_{i},x_{i-2}\right)\ldots ,f\left(x_{i},x_{i-n}\right)\right)=\left(f\left(\Delta x_{-1}\right),f\left(\Delta x_{-2}\right),\ldots ,f\left(\Delta x_{-n}\right)\right),$$

where by $f(x_{i},x_{i-j})$ we describe the price change between the *i* and *j* reading; for which we obviously observe the tendency that the Δ value will increase, while we move away from the initial element in the time series. We use the following formula to derive the symbolic representation for the data:

(9) $$f\left(\Delta \left(x_{i}\right)\right)=\left\{\begin{array}{c} k\mathrm{if}\left(2\cdot k+1\right)\cdot s\leq \Delta x_{i}\\ \ldots \\ 1\mathrm{if}s\leq \Delta x_{i}<3\cdot s\\ 0\mathrm{if}|\Delta x_{i}|\leq s,\mathrm{where}s=\bar{\Delta x_{-1}}\\ -1\;\mathrm{if}-s\leq \Delta x_{i}<3\cdot s\\ \ldots \\ -k\mathrm{if}\Delta x_{i}<-\left(2\cdot k+1\right)\cdot s \end{array}\right.$$

In general, the whole range, within which every analyzed value (original or relative) could be found was divided into equally-sized intervals. Every interval had some predefined value (or symbol), which was used instead of this value. Thus, for any value in range 〈−2.0;−3.0〉, we used the representative equal to −3, while for the −3.01 value, we would use the representative equal to −4, and so on.

**Figure 4:**
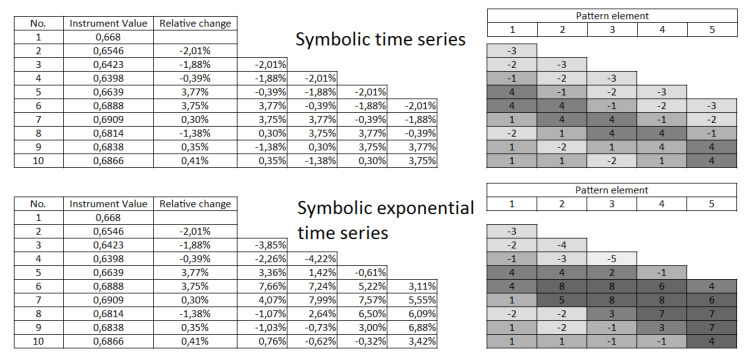
Comparison of the symbolic time series and symbolic exponential time series representation.

This concept was originally introduced in [37]. The main difference between the approach introduced in this article and the concept derived in the above work was the relative value calculation (called here as the exponential symbolic time series). The present approach (denoted further as the symbolic time series) took into account the difference between the two neighboring values; while the exponential symbolic time series calculated the difference between the actual price value and the first price value observed in the analyzed time series. The summary of both approaches can be found in Figure 4.

Transforming data into a symbolic form is one of the tools used in the study of dynamic systems [38]. The aim of this operation is to provide a simplified picture of complicated dynamics that ensures the preservation of the most important features of the tested object while enabling the use of new methods and accelerating or simplifying calculations. It is especially useful for nonlinear and chaotic time series [39]. Symbolization is based on dividing the state space of the examined system into a finite number of elements and describing the trajectories of individual points in accordance with this division [14]. Symbolization means describing an original time series with a set of symbols from the established alphabet. A number of symbols may vary from merely two (like “ups” and “downs” in [12] or “0” and “1” in [38]) to a few, depending on the objectives of the study.

In general, we introduce symbolic description when:

- we need dimensionality reduction;
- noise filtering is needed;
- particular symbols have a special meaning for the final outcome and interpretation;
- we want to use additional text measures or data mining methods.

Introducing a symbolic description of time series raises the problem of equal values [3]. Originally, for time series with continuous values, Brandt and Pompe suggested adding a small random perturbation in this case, but while we worked with a symbolic description, this did not apply. In our research, we assumed that if $x_{i}=x_{j}$, then $x_{i}<x_{j}$ if i<j; however, also other methods have been proposed [40,41]. In the case of PE, introducing symbolic description aims at providing a more precise description of the time series.

## 4. Experiments

The goal of the conducted experiments was the entropy analysis for the currency pairs. We investigated the impact of the data representation on the entropy value. In the experiments, we analyzed the original currency pairs’ data, relative values (calculated according to the approach presented in the previous section), and a few different symbolic values’ representation related to the length of the description; in this context, by the length, we understand the larger number of past values used in the symbolic description.

Having many time series, the most frequent challenge is their clustering and classification. In this case, the entropy of the entire time series was calculated and used for further purposes. In the case of financial data, the more important task was finding patterns and predictions. Because our main focus was the variability of the permutation entropy based on different ordinal patterns’ constructions, we focused separately on the analysis of the histograms for all entropy values; dependent on the selected data representation, as well as the variability of the permutation entropy itself. In this case, the typical technique is moving-window analysis. Entropy was calculated for each window separately and investigating how entropy changed over time in relation to the original financial time series.

At the same time, due to a large number of symbolic representation variants, discretization levels, parameters related to entropy, and other parameters, we were forced to limit the data presented in the further part of this section.

### 4.1. Experiments Design

All experiments were conducted on four different currency pairs:

- EURUSD,
- GBPUSD,
- USDCAD,
- USDJPY.

Each single currency pair included 2500 values, where every single value (reading on the chart) was generated at the end of the daily session. Thus, the overall analyzed period covered approximately 10 years: from June 2007 to July 2017. The above data and time period were selected due to the good availability of high-quality data free from missing values or outliers. Moreover, a selected time period not only covered the different kinds of trends on the market and financial crisis (2008–2009), but also allowed us to investigate if the proposed entropy-based approach was capable of delivering good-quality information in the case where the situation on the market was not stable.

In all experiments for every currency pair, the permutation entropy was calculated. In this section, we present the results for the time window equal to 30 readings and n=4. There is a natural 5 day (a week) period in the analysis of the financial time series [42]. Hence, in our research, we set n = 4, which corresponded to 5 readings of absolute values in a series (one week), and the time window was 30, as the minimum multiple of 5 (multiple full-week-value) that met the permutation entropy calculation requirements for n=4. Such parameters allowed calculating the entropy value for the 4 elements on the basis of the 27 values in every time window. This met the condition related to the entropy calculation, where every pattern (for n = 4, there were overall n!=24 patterns) had the possibility of being observed. For the forex financial data, this allowed analyzing exactly a 6 week period, which would be considered rather as a long-term investment, allowing minimizing the possible random noise on the market as much as possible. Thus, on the one hand, these parameters met the conditions for calculating PE, and on the other hand, they corresponded to the periods of analysis of financial time series used by investors.

All presented results applied for all time windows (almost 2500 windows for every currency pair). Thus, the first time window included readings starting at 1 reading up to 30; the second window started at 2 and ended at 31, and so on. At the same time, the permutation entropy was calculated for 4 successive elements, thus 1..4, 2..5, 3..6, and so on, ending at 27..30, which gave us 27 (possibly different) values in every time window.

In all our experiments, we used the Dn to mark that the data were discretized with the number of discrete values equal to *n*. The notation of our data is as follows:

- Original data: data without any initial preprocessing;
- Relative data: includes the relative differences between two successive price readings;
- Symbolic series *k*: every single reading consists of a number of past readings as well. Each element is discretized (on the basis of relative data);
- Symbolic exp series: the symbolic exponential time series is built on the basis of the equations presented in Section 3.

### 4.2. Entropy Variability Analysis

Experiments related to the entropy analysis for the successive time windows were used to evaluate the impact of the financial data representation on the entropy values. To achieve these goals, we performed the detailed analysis separately for every single currency pair. Moreover, the charts were divided into two parts:

- Original data, relative data, and Symbolic Series 4 with Discretization 7;
- Symbolic Series 3, 4, and 5 (Discretization 5 and 7).

We investigated what was the impact of the proposed symbolic time series representation on the entropy values (in this particular case, we limited the number of elements included in the symbolic description to 4). At the same time, we compared the different discretization levels (5 and 7) strictly for the symbolic series representation.

**Figure 5:**
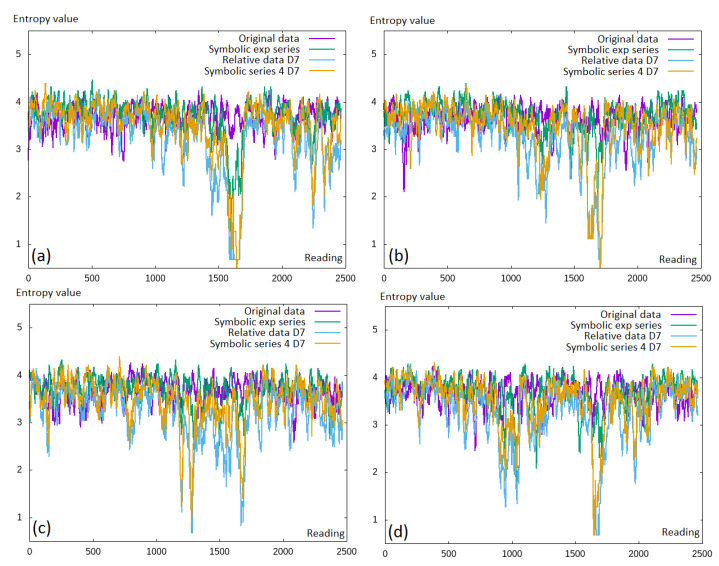
Permutation entropy values for the full time window including approximately 2500 values: (**a**) EURUSD; (**b**) GBPUSD; (**c**) USDCAD; (**d**) USDJPY. exp, exponential.

In the first part of the experiments, we analyzed the differences in the entropy obtained with the use of the original data, relative data, symbolic exponential data, and the symbolic series. This was repeated for all four currency pairs and can be seen in Figure 5. The expected entropy values should be as low as possible, which meant that some additional information was obtained. It seemed that there was little (or none at all) difference between the original financial data (in purple) and the symbolic exponential series (the green line); while for the calculation of the relative, discretized values made the entropy reduction especially visible in the middle of the charts.

**Figure 6:**
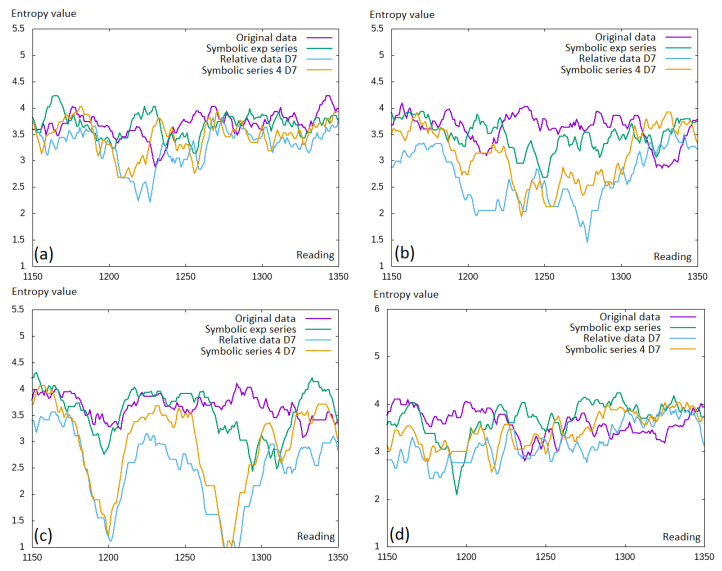
Permutation entropy values for the selected time window fragment. Comparison of the original data, symbolic exponential data, relative data, and symbolic series (**a**) EURUSD; (**b**) GBPUSD; (**c**) USDCAD; (**d**) USDJPY.

A small fragment of the data was selected to better capture this observation (see Figure 6). Here, we can see that actually, the entropy was decreased for the discretized values. This, in general, was compatible with the intuition that moving towards the discretized values, some information was lost.

**Figure 7:**
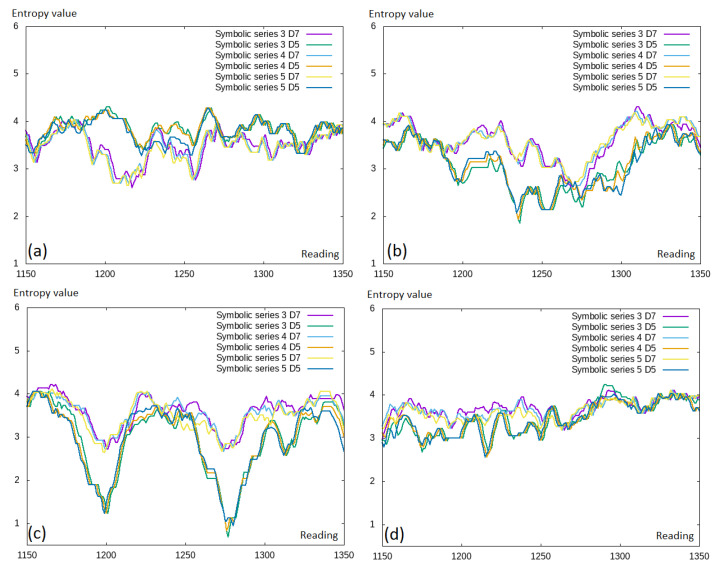
Permutation entropy values for the selected time window fragment. Comparison of the different symbolic series: (**a**) EURUSD; (**b**) GBPUSD; (**c**) USDCAD; (**d**) USDJPY.

However, by extending the symbolic representation, some additional information was added; thus, the differences in the entropy values between the symbolic representation of different lengths should differ. We investigated this in Figure 7, where we can see the comparison of the symbolic representation with different numbers of symbols included and different discretization levels. These results were counter-intuitive; thus, we would rather expect that the larger number of symbols included would lead to higher entropy values. We observed different entropy levels between different discretization levels (D5 and D7); however, there was no visible difference between the symbolic representation length.

### 4.3. Entropy Histograms

For the analysis of the entropy distribution for the whole analyzed time window, entropy histograms were calculated. In the case of the developed experiments, entropy had the following range: 0.0, which meant no entropy at all, up to almost 4.6, which was understood as the maximal possible disorder calculated as the log(n!) (with a base = 2), where n=4. Thus, the entropy calculated for the whole time window was grouped in the ranges with a difference of 0.1 between two separate intervals, which gave us 47 intervals: (0.0–0.1], (0.1–0.2], and so on.

**Figure 8:**
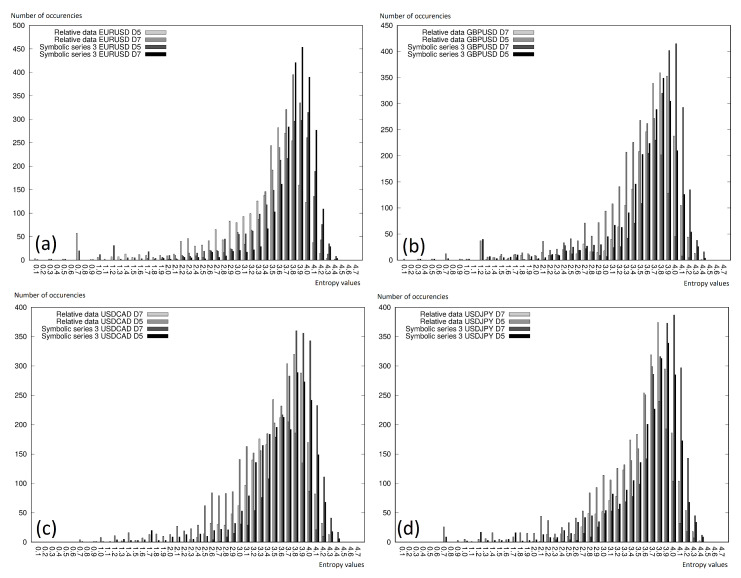
Permutation entropy histograms for the selected time window fragment. Comparison of the relative data and symbolic series: (**a**) EURUSD; (**b**) GBPUSD; (**c**) USDCAD; (**d**) USDJPY.

**Figure 9:**
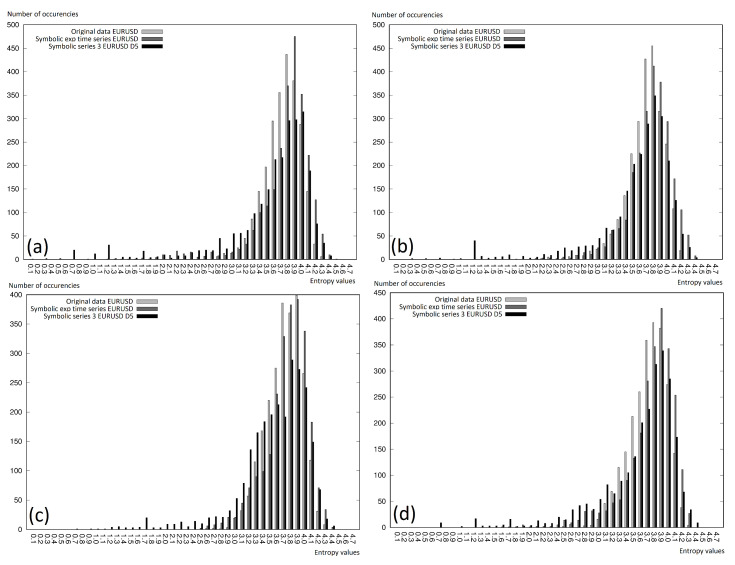
Permutation entropy histograms for the selected time window fragment. Comparison of the original data, symbolic exponential data, and symbolic series: (**a**) EURUSD; (**b**) GBPUSD; (**c**) USDCAD; (**d**) USDJPY.

On the charts in Figure 8 and Figure 9, we can see the entropy histograms, where the vertical axis represents the entropy value, which fit in the given interval, while the horizontal axis represents the deterministically calculated intervals. The figures were generated for every currency pair separately and included two different analyses:

- relative data with D5 and D7 discretization levels and Symbolic Series 3 with D5 and D7 discretization levels (Figure 8);
- original data, symbolic exponential, and Symbolic Series 3 (with discretization level D5); see Figure 9.

We can conclude that the lower entropy was observed for the cases where the more elements were observed in the intervals. Very often, a right-shifted normal distribution was observed. Thus, it is worth mentioning that using the symbolic representation did not affect the differences in the entropy (in comparison to the original data). This was especially interesting, because of the fact that the symbolic representation showed the information not only about the present reading, but also involved the information given in the previous readings. The information extension affected its higher uniqueness, and the histograms presented on the charts in this section clearly showed that the growth of the information in the case of the symbolic representation did not visibly change the entropy.

### 4.4. Statistical Tests

Statistical tests were designed on the basis of the means of a non-parametric statistical hypothesis test: the Friedman test for α=0.05. Tests were conducted for the entropy values grouped in the exact same manner, as was observed in the case of the histograms presented in the previous subsection. The goal of these tests was to evaluate if there existed a statistical difference between the symbolic data representation and other price representations. Statistical tests were performed for all currency pairs (jointly); however, we divided this procedure into two separate analyses:

- comparison of mean ranks between relative data, Symbolic Series 3, Symbolic Series 4, and Symbolic Series 5, in every case, the discretization level was equal to D5. These results are presented in Table 1;
- comparison of mean ranks between original data, symbolic exponential series, and Symbolic Series 3 (with discretization level equal D5). These results are presented in Table 2.

For the first analysis presented in Table 1, the Friedman test parameters were as follows: Chi-squared = 30.96, degrees of freedom = 3, and 5% critical difference = 0.221796. The hypotheses for the comparison across repeated observations were as follows:

- H0: the distributions were the same across repeated observations;
- H1: the distributions across repeated observations were different.

Statistical tests indicated that between the histograms’ (for all currency pairs presented jointly) relative data and all three symbolic series (with the discretization equal D5), there was a statistical difference; while there was no statistical difference between Symbolic Series 3, 4, and 5. Thus, Hypothesis H0 was rejected, while hypothesis H1 was confirmed.

For the analysis presented in Table 2, the Friedman test parameters were as follows: Chi-squared = 89.29, degrees of freedom = 2, and 5% critical difference = 0.151787. The hypotheses for the comparison across repeated observations were as follows:

- H0: the distributions were the same across repeated observations;
- H1: the distributions across repeated observations were different.

The statistical tests indicated that it was possible to reject the hypothesis H0 and to confirm the hypothesis H1. From the comparison of all representations: original data, symbolic exponential series, and Symbolic Series 3, there were statistical differences; thus, the histograms for all these approaches were different.

We also conducted statistical tests, the main goal of which was to estimate which method statistically would have the lowest entropy value for all analyses. To achieve this goal, we used all collected entropy values for every method, which had an overall 9868 analyzed readings for every method. In Table 3, the Friedman test parameters are as follows: Chi-squared = 914.02, degrees of freedom = 2, and 5% critical difference = 0.027186. Such low values of the critical difference were related mostly to the number of analyzed readings (reaching almost 10,000).

In this case, the tests were used mostly to calculate the mean ranks for the sample. On this basis, it was observed that the best rank, equal to 1.79755, was obtained in the case of Symbolic Series 3 (with the discretization level D5). It was better than the rank for the original data by 0.18, and it was equal to 1.9775 (and better than the rank for the symbolic exponential series by 0.4276 and equal to 2.2251). Such large differences, with the 5% critical difference equal to 0.0272, meant that Symbolic Series 3 in comparison to the original data and symbolic exponential series had not only the highest rank, but was also critically better in comparison to these approaches for the entropy values for all possible readings.

## 5. Discussion

The results of the numerical experiments from the previous section could be interpreted in two ways: in terms of assessing market measures and in terms of further possibilities of using entropy in market analysis.

Due to the large number of free parameters existing in the proposed symbolic representations, it was very difficult to point out their best values, which visibly affected the quality of the results. Thus, our motivation was to find a way to estimate what was the impact of these parameters on the information acquired from the market. Permutation entropy could significantly expand the set of technical analysis tools and even become the basis for the construction of new ones. However, the entropy value itself gave us information about the volatility on the market, where the information was related only to the price movement, and not the direction of the changes.

It was obvious that using over-simplified discretization would lead to visible information loss. However, what was interesting was that in this situation, this was not true in the opposite way. Thus, it seemed unlikely that extending the symbolic representation over some point would not positively affect the amount of information derived for the decision-maker. The unnecessarily complicated representation not only did not include more information (no visible differences in the entropy values), but also led to an over-complicated description of the market situation that was difficult to analyze by the decision-maker. This matters both in the case of fully automatic transaction systems or high-frequency transaction systems based on patterns/symbolic representation, and even more in the case of human-led analysis (investor or expert). In both cases, it is important that at a certain information level, the form of information should be as simple as possible.

The key aspect from the point of view of the decision-maker is the trade-off between the quality of information and the degree of difficulty of the observed information. In the case where a fast reaction is needed, the greater deviation of the optimal decision is acceptable.

The results achieved also raise the question about the influence of the different descriptions on prediction, which is the most important issue for all investors. Permutation entropy gave us information about the potential volatility on the market and additionally how good (or how bad) the tools used by the decision-maker were. The interpretation of the entropy was as follows: in the case of the small entropy, the conviction of the quality of signals generated by tools/indicators should be large. In the opposite situation, the decision-maker should have limited trust in the observed signals. Please note that the combination of entropy and some selected market indicators (or patterns) could have some predictive power and could be measured in terms of the quality of signals derived for the decision-maker.

**Figure 10:**
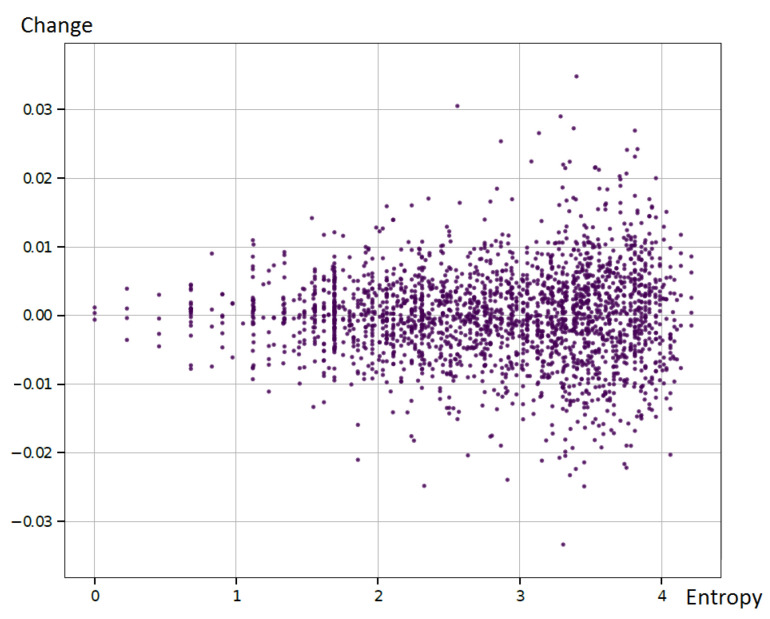
Example correlation between the entropy value and the relative change of the instrument between the actual and the next reading for the EURUSD symbolic exponential series.

To evaluate this observation, we used the selected currency pair and its symbolic representation (as described in this article) and estimated the future price change for every observed entropy value. We used the assumption that the value of the instrument greater than that observed in the actual reading was equal to the BUYsignal, while the opposite situation was the SELLsignal. The example results are presented in Figure 10.

As the distribution was almost symmetrical in relation to zero, it was clear that the price changing direction (rising or falling) was not correlated with the entropy values. On the other side, it could be seen that it was correlated with the standard deviation of the changes. Thus, the small entropy was related to the small standard deviation of the price change on the market. This observation corresponded to the financial Markowitz model introduced by H.Markowitz in [43], where the risk of the investment was presented by the standard deviation. In other words, a large standard deviation corresponded to a large risk from the investment.

To sum up, we were not able to clearly indicate that some particular entropy values could be used to predict the exact price direction. However, it was possible to show, for example, that large entropy values were related to the large standard deviation, which was considered as a risk measure; thus, large entropy meant large risk. This initial observation prompts undertaking experiments on a larger number of cases that will confirm or reject the hypothesis about the usefulness of entropy also as a predictor of financial market volatility.

## 6. Conclusions

In this paper, we investigated the impact of the various symbolic representations of the financial data on the permutation entropy values. It is a common assumption that in the case of the discretization process and for the symbolic representation, the noise observed on the market is reduced. However, at the same time, we observed some information loss. This observation was confirmed in the first part of the experiments. Interesting results were observed in the case of the analysis of different symbolic time series. It was rather counter-intuitive that in the case of extending the symbolic representation by new elements, the entropy values should change as well. In the presented approach, we observed obvious differences between entropy values for the different discretization levels, while the symbolic representation length itself did not affect the entropy values. We derived a simple method to transform the financial data into the symbolic representation. Permutation entropy could be used as a tool to estimate the information gain/information loss between different financial instrument representations.

The obtained results confirmed rather an obvious conclusion that introducing the symbolic data representation led to information loss. However, the second conclusion related to the symbolic data representation itself was rather counter-intuitive, since extending the symbolic time series used in the description did not affect the permutation entropy values. It seemed that for the presented financial data from the forex market, introducing the symbolic representation or selecting the different discretization parameters affected the entropy values, while the extension of the symbolic representation was less relevant.

## 7. Future Works

Our previous research was focused on deriving different, simplified ways to describe the situation on the market. The discussed symbolic representation could be used not only for the price itself, but also for the market indicators. This gives us the opportunity to work on the multidimensional symbolic time series in the future. Thus, we could not only simplify the price itself, but also the whole trading system, including some number of market indicators.

The second direction of our future work is focused on deriving a simple and effective way to evaluate the quality of symbolic representation. At this moment, most systems are evaluated on the basis of the comparison between the expected and actual price direction. The range of this movement is not included. Moreover, trading systems on the forex market assume the single-instrument investments, where building the currency pair portfolio is an obvious direction.

## Figures and Tables

**Table 1:** Friedman test results and differences of the mean ranks (rel. data, relative data; symb. s. *k*, symbolic series of length *k*).

Friedman Test				
Chi-squared	30.955213			
degrees of freedom	3			
*p*-value is less than	0.0001			
5% critical difference	0.221796			
**The Differences of the Mean Ranks**				
	**rel. data**	**symb. s. 3**	**symb. s. 4**	**symb. s. 5**
rel. data	0.0000	0.4894	0.5505	0.5346
symb. s. 3	0.4894	0.0000	0.0612	0.0452
symb. s. 4	0.5505	0.0612	0.0000	0.0160
symb. s. 5	0.5346	0.0452	0.0160	0.0000

**Table 2:** Friedman test results and differences of the mean ranks (org. data, original data; sym. exp., symbolic exponential series; symb. s. *k*, symbolic series of length *k*).

Friedman Test			
Chi-squared	89.287523		
degrees of freedom	2		
*p*-value is less than	0.0001		
5% critical difference	1.542553		
**The Differences of the Mean Ranks**			
	**org. data**	**sym. exp.**	**symb. s. 3**
org. data	0.0000	0.2660	0.8191
sym. exp.	0.2660	0.0000	0.5532
symb. s. 3	0.8191	0.5532	0.0000

**Table 3:** Friedman test results and mean ranks (the best rank is indicated by the bold font).

Friedman Test	
Chi-squared	914.018736
degrees of freedom	2
*p*-value is less than	0.0001
5% critical difference	0.027186
**Mean Ranks**	
original data	1.9774523713
symbolic exponential	2.2250709364
symbolic series 3	**1.7974766923**
